# Dynamics of Physical Interaction between HIV-1 Nef and ASK1: Identifying the Interacting Motif(S)

**DOI:** 10.1371/journal.pone.0067586

**Published:** 2013-06-14

**Authors:** Balawant Kumar, Chakrapani Tripathi, Ranjana K. Kanchan, Jitendra Kumar Tripathi, Jimut K. Ghosh, Ravishankar Ramachandran, Smrati Bhadauria, Raj Kamal Tripathi

**Affiliations:** 1 Division of Toxicology, Central Drug Research Institute (Council of Scientific & Industrial Research), BS-10/1, Sector-10 Jankipuram Extension, Uttar Pradesh, India; 2 Division of Molecular and Structural Biology, Central Drug Research Institute (Council of Scientific & Industrial Research), BS-10/1, Sector-10 Jankipuram Extension, Uttar Pradesh, India; Hertie Institute for Clinical Brain Research and German Center for Neurodegenerative Diseases, Germany

## Abstract

FasL mediated preferential apoptosis of bystander CTLs while protection of infected CD4^+^T cells remains one of the hallmarks of immune evasion during HIV infection. The property of infected host cells to evade cell-autonomous apoptosis emanates from ability of HIV-1Nef -protein to physically interact with ASK-1 and thereby inhibit its enzymatic activity. The specific domains of HIV-1Nef through which it may interact with ASK1 and thereby impair the ASK1 activity remain unidentified so far and represent a major challenge towards developing clear understanding about the dynamics of this interaction. Using mammalian two hybrid screen in association with site directed mutagenesis and competitive inhibitor peptides, we identified constituent minimal essential domain (152 DEVGEANN 159) through which HIV-1Nef interacts with ASK1 and inhibits its function. Furthermore our study also unravels a novel alternate mechanism underlying HIV-1 Nef mediated ASK1 functional modulation, wherein by potentiating the inhibitory ser^967^ phosphorylation of ASK1, HIV-1Nef negatively modulated ASK1function.

## Introduction

The human immunodeficiency virus1 (HIV-1) Nef, a 27 kDa accessory protein has a preeminent role in viral replication and *in vivo* pathogenesis as evident by undetectable viral loads and absence of clinical manifestation of AIDS in patients infected with HIV-1Nef defective virus [1-5]. Eradication of host immune system by way of gradual depletion of T lymphocytes [6,7] remains the most steady pathogenic feature of HIV-1 infection. Non-infected bystander CD8^+^ cells undergo apoptosis, while infected cells remain protected [8-10]. Increased killing of bystander CD8^+^ cells is mediated in part through HIV-1Nef induced Fas ligand (FasL) up regulation, on the surface of the virally infected T cells [11,12]. The subsequent interaction of FasL with Fas (CD95) displayed on neighboring HIV-1 infected cells, T lymphocytes may lead to bystander cell killing and thus form an important mechanism of immune evasion [13]. Since virally infected cells that exhibit Nef induced up regulation of FasL, also express the cognate receptor i.e. Fas (CD95), the possibility of rapid cell-autonomous apoptosis of the infected cells mediated through FasL/Fas cis-ligation becomes much obvious [14,15]. Similarly, interaction of membrane bound TNF-α on macrophages with TNF-α receptor present on infected cells may also elicit apoptosis in infected cells [16,17]. However, unlike bystander cells, HIV-1 infected cells readily evade progression into apoptotic cascade. This survival advantage is conferred upon HIV-1 infected cells by Nef through its ability to modify intracellular milieu by interacting and inhibiting apoptosis signal-regulating kinase (ASK1) leading to development of resistance towards FasL/ TNF-α induced apoptosis. Apoptosis signal-regulating kinase 1 (ASK1), a 151-kDa serine/threonine protein kinase, is a member of the MAPK-Kinase family and activates both p38 and JNK1 pathways by directly phosphorylating and activating SEK1 [18-20]. It represents a key signaling node in the FasL/ TNF-α mediated death-signaling pathway [21]. Proposed mechanism underlying functional regulation of ASK1 activity includes binding of factors TRAF2 [22] and dissociation of inhibitors 14-3-3, Hsp70, glutaredoxin-1 and thioredoxin (TRX) [23-26]. Ample experimental evidence exist in support of ability of Nef to simulate the action of ASK-1 negative regulator/s. Association of Nef-ASK1 leading to impairment of ASK1 pro-apoptotic function in HIV-1 infected cells leading to dephosphorylation of JNK1/p38 kinase in TNF-α induced cells is well documented. This action of Nef while on one hand allows virally infected host cells to successfully evade host immune response by acquiring resistance to FasL / TNF-α mediated apoptosis, paradoxically on the other hand potentiates the localized destruction of HIV-1 specific cytotoxic T cells and bystander cells which are attempting to mediate viral clearance. The prolonged survival owing to Nef/ASK-1 interaction allows the HIV-1 infected host cells to produce new infectious virons leading to increased viral load. By playing a decisive role in disease progression, the ASK1-Nef interaction acquires paramount significance as a possible target for therapeutic intervention. Knowledge regarding the precise dynamics of this interaction could provide molecular basis for understanding the immune evasion mechanism by Nef. In view of this, the current study was planned so as to identify the critical domains with in ASK1 and Nef whose interaction regulate death receptor mediated apoptosis.

## Results

### Identification of the ASK1 Regions That Interact with Nef

Interaction of Nef protein with host protein is largely governed by its unique structural attributes. These structural attributes and other post translational modifications of Nef are maintained in mammalian cells. Therefore we adopted mammalian two hybrid system in which Nef and host proteins were cloned in vectors to study protein interaction. This model mimics the interaction as would happen in HIV 1 infected cells.

**Figure 1:**
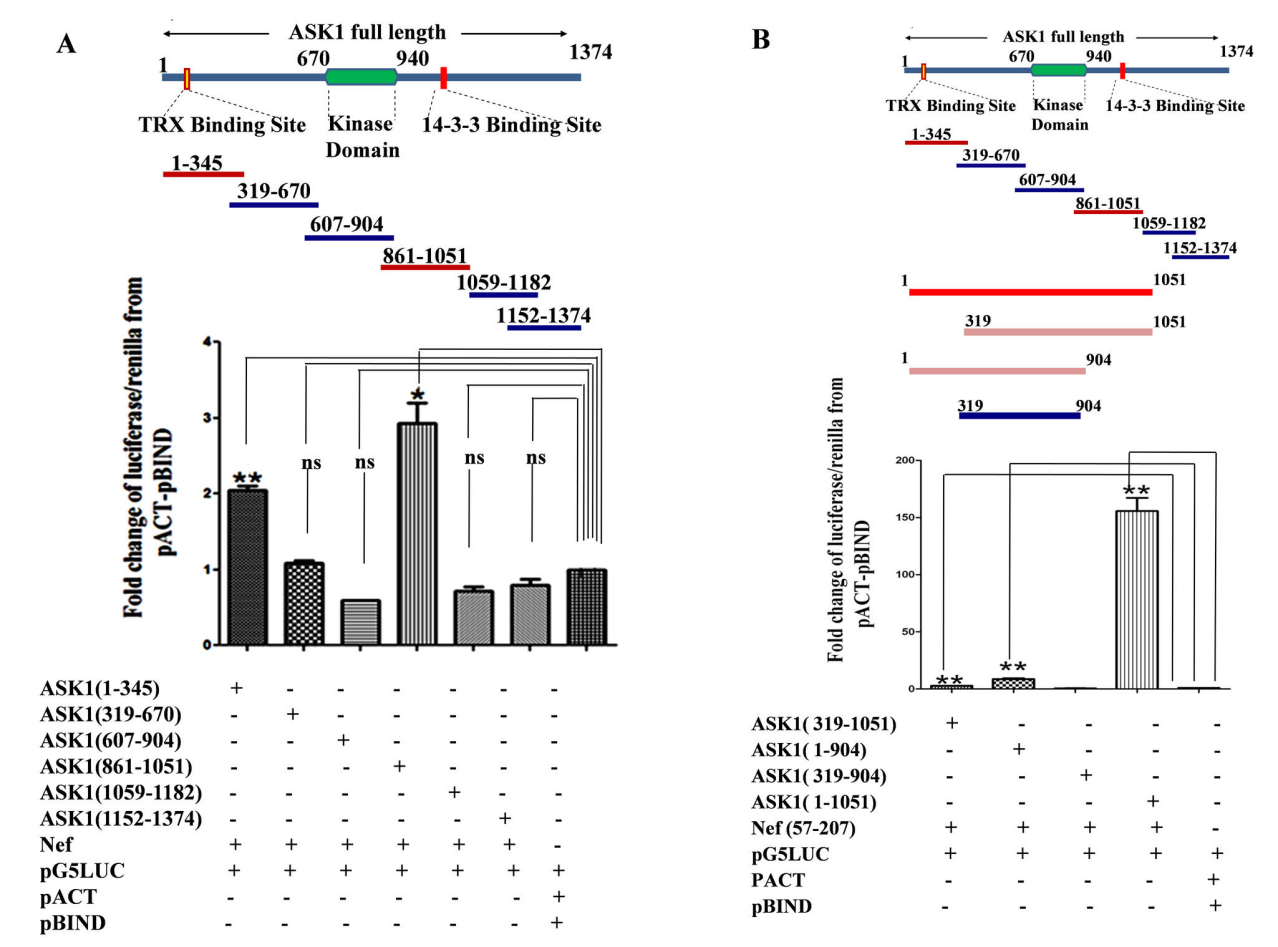
Identification of Nef interacting region within ASK1. (**A**) ASK1 overlapping fragments casing full length of ASK1 were designed to generate multiple ASK1 truncations viz. ASK1 (1-345 aa), ASK1 (319-670 aa), ASK1 (607-904 aa), ASK1 (861-1051 aa), ASK1 (1059-1182 aa), ASK1 (1152-1374 aa). The HEK-293 cells were co-transfected with ASK1 truncations and Nef along with pG5*LUC* reported vector. Different truncations exhibited varying degrees of interaction as measured by fold increase in luciferase/renilla expression over pACT-pBIND negative control vector viz. 2.05, 1.08, 0.59, 2.93, 0.71, 0.79 respectively. ASK1(1-345 aa) and ASK1(861-1051 aa) fragment showing significant interaction while other fragments are not significantly (ns) interact with Nef (**B**) A distinct ASK1 truncation that contained only C-terminal Nef interacting domain and lacked N-terminal Nef interacting domain i.e. ASK1 (319-1051 aa) was designed. Similarly, another distinct ASK1 truncation that contained only N-terminal Nef interacting domain and lacked C-terminal Nef interacting domain i.e. ASK1 (1-904 aa) was also designed. Using co-transfection studies in HEK-293 cells, both the truncations were individually evaluated for their interaction ability with Nef vis a vis ASK1 (1-1051 aa) truncation that harbored both N and C terminal domains and ASK1 (319-904 aa) truncation that lacked both N and C terminal domains. Co-transfection with ASK1 (1-1051 aa) caused 156.56 fold increase in luciferase/renilla expression compared to negative control pACT-pBIND while other ASK1 fragment viz. ASK1 (319-1051 aa), ASK1 (1-904 aa), ASK1 (319-904 aa) caused only 2.83, 8.75, 0.59 fold increases respectively. The data are means +SEM from three distinct experiments. *P* values of <0.05 were obtained (two-tailed paired *t* test) when luciferase/renilla expression from ASK1 fragments and Nef co-transfected cell were compared with negative control pACT-pBIND.

HEK-293 cells were co-transfected with vectors coding for a series of overlapping GAL4-ASK1 truncations ASK1 1-345 amino acid (aa), ASK1 (319-670 aa), ASK1 (607-904 aa), ASK1 (861-1051 aa), ASK1 (1059-1182 aa), ASK1 (1152-1374 aa) (Figure 1A) and VP16-Nef, along with pG5*luc* vector. Luciferase reporter assay was performed 48 hrs post transfection. Our results showed that compared to pACT-pBIND (negative control), the cells co-transfected with Nef (57-207 aa) and ASK1 (1-345 aa) fragment or ASK1 (861-1051 aa) fragment showed 2.05 and 2.93 fold increase in luciferase/renilla expression respectively. In contrast the cells transfected with other ASK1 truncations showed much less luciferase/renilla expression. Collectively these results indicate that within ASK1 protein fragment 1-345 aa (N terminal) and 861-1051 aa (towards C- terminal) regions contributed significantly in the interaction with Nef. Experiments were done three times and all data was analyzed by graph-pad prism 5.

### Deciphering minimal ASK1 region essential for interaction

Our next objective was to deduce as to how much these two regions of ASK1 (1-345 aa and 861-1051 aa) contribute towards overall affinity of ASK1 for Nef. To address this, we cloned different truncations of ASK1, with or without these regions in accordance to scheme depicted in Figure 1B. As shown in Figure 1B, compared to pBIND-pACT negative vector control, the cells harboring ASK1 (1-1051 aa) fragment which contained both the interacting regions (1-345 aa and 861-1051 aa) showed 162 fold increases in luciferase/renilla expression. Cells infected with truncated fragment (319-1051 aa) of ASK-1 that was devoid of N terminal interacting region (1-318 aa) showed 2.9 fold increases in luciferase/renilla as compared to pACT-pBIND negative vector. This indicated that deletion of N-terminal (1-318 aa) from ASK1 (1-1051 aa) results in markedly decreased Nef protein’s interaction affinity for ASK1 (319-1051 aa). Further on, results indicated that 1-318 aa of ASK1 (1-1051 aa) is important for its interaction with Nef. Moreover, cells co-transfected with Nef and ASK1 (1-904 aa) fragment that harbored 1-318 aa region but not 905-1051 aa region, showed 9.15 fold increase in luciferase/renilla compared to pACT-pBIND negative control (Figure 1B). This shows that on deletion of C- terminal 905-1051 aa from ASK1 (1-1051 aa) region, the ability of ASK1 (1-904 aa) to interact with Nef was markedly impaired. Result showed that ASK1 regions corresponding to 319-904 aa did not interact at all with Nef as evident by no change in the luciferase/renilla expression compared to negative control. Collectively these results indicated that not only N-terminal (1-318 aa) but C-terminal (905-1051 aa) of ASK1 was also important for its interaction with Nef and that addition the two minimal interacting regions of ASK1 (1-318 aa and 905-1051 aa) to the otherwise non interacting region (319-904 aa) render it capable of interacting with Nef. The whole ASK1 (1-1051 aa) fragment was identified as minimal essential region for its interaction with HIV-1Nef. Both fragments viz. ASK1 (1-318 aa) and (905-1051 aa) substantially increase the affinity of ASK1 for Nef. Interestingly, both these regions i.e N-terminal ASK1 (1-318 aa) and C-terminal (905-1051 aa) harbored binding sites for two upstream negative modulators viz. Thioredoxin and 14-3-3 protein respectively.

### Prediction of Nef domain that interact with minimal ASK-1 regions:

**Figure 2:**
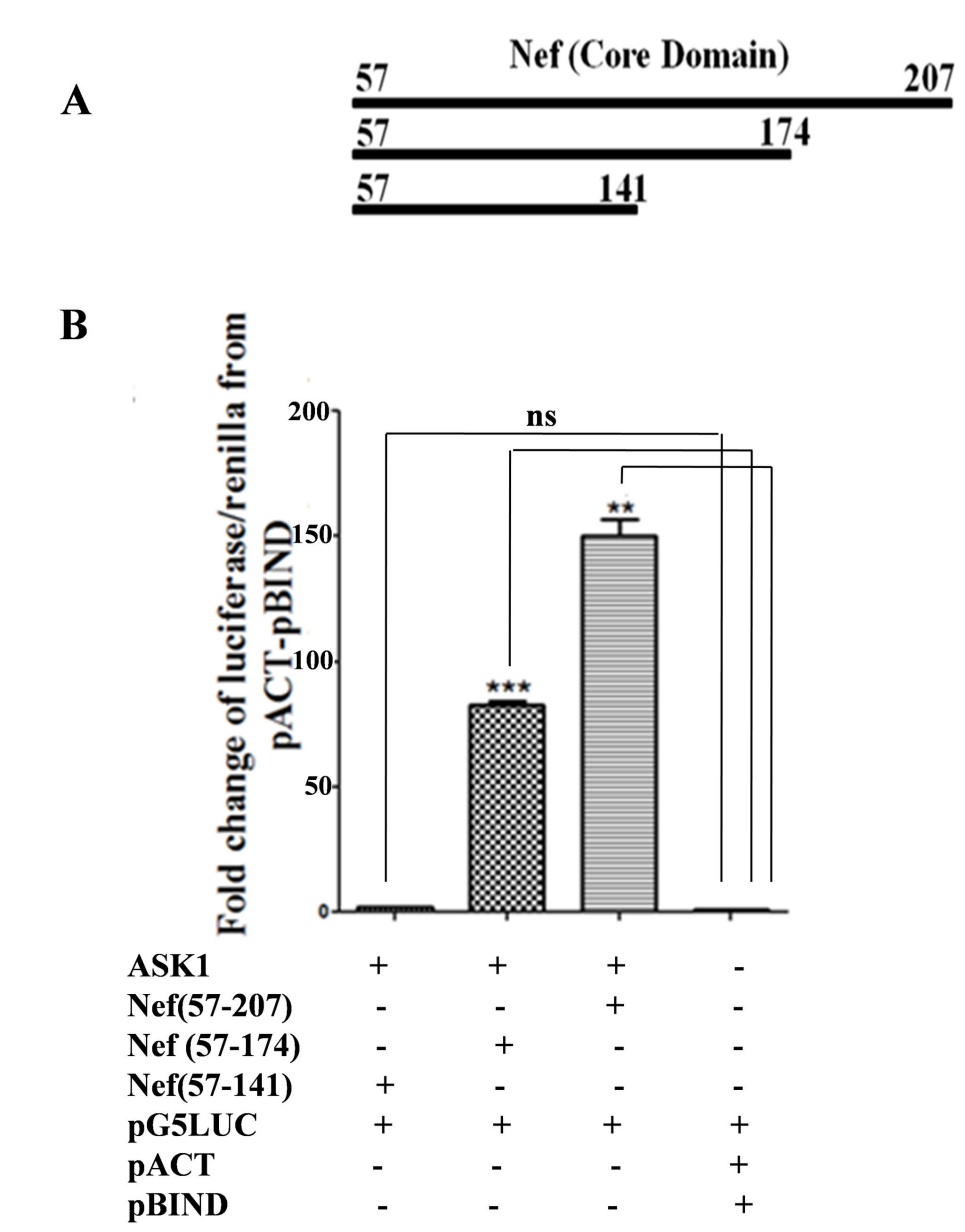
Identification of Nef region interacting with ASK1. (**A**) Using Nef core domain (57-207 aa) sequences, two distinct Nef truncations were designed for mammalian two hybrid studies by successively deleting 33 aa form C-terminal and subsequent cloning in pACT vector so as to obtain Nef (57-174 aa), Nef (57-141 aa) fragments. The Nef core domain (57-207 aa) served as positive control. (**B**) The HEK-293 cells when co-transfected with ASK1 (1-1051aa) fragment and either of Nef fragments viz. Nef (57-207 aa), Nef (57-174 aa), Nef (57-141 aa) and pG5*LUC* vector showed 152.56 ,81.68 ,and 1.9 fold increase in expression of luciferase/renilla over pACT-pBIND negative control vector respectively. The data are means +SEM from three distinct experiments. *P* values of <0.05 were obtained (two-tailed paired *t* test) when luciferase/renilla expression from ASK1 fragments and Nef co-transfected cell were compared with negative control pACT-pBIND.

Having identified Nef interacting minimal essential regions with in ASK1, our next objective was to identify the critical regions within Nef that interacted with ASK1 (1-1051 aa). Since Nef exhibits structure based interaction, therefore deletion was made from c-terminal generating truncated variants of Nef protein that had altered structure. These truncation were made by deleting 33 aa and 66 aa from C-terminal to obtain two Nef variants viz. Nef (57-174 aa), Nef (57-141 aa) respectively (Figure 2A). Nef core domain (57-207 aa) which served as reference, exhibited a 152.56 fold increase in luciferase/renilla as compared to pACT-pBIND vector control. Interestingly the two truncated fragments viz. Nef (57-174 aa) and Nef (57-141 aa) exhibited 81.68, 1.90 fold increase in luciferase/renilla compared to pACT-pBIND vector control respectively. These results indicated that deletion of 33 aa from C-terminal of Nef (57-174 aa) caused considerable decrease in the interaction affinity of Nef with ASK1 (1-1051 aa) and deletion of further 33 aa from Nef (57-141 aa) caused complete loss of interaction with ASK1 (1-1051 aa). The result indicated that C-terminal of Nef (141-207 aa) contained critical domain that allowed Nef to interact with ASK1 (1-1051 aa).

### Molecular mechanisms up and downstream to ASK1: Deregulation due to Nef-minimal ASK1 interaction:

Nef associated apoptotic deregulation in infected cells is attributable to ability of Nef to physically interact with and thereby functionally modulate ASK1 activity by interfering with upstream (Thioredoxin) and downstream cascades (p38/JNK1) [22,26]. In our study also the, identified ASK1 critical regions (N-terminal: 1-318 aa and C-terminal: 905-1051 aa) contained residues through which upstream modulators viz, Thioredoxin and 14-3-3 exerted regulatory control over ASK1.Therefore in current study we studied dynamics of Nef-minimal ASK1 physical interaction with special reference to critical upstream and downstream events that relay death signal to/from ASK1 and thus represent validated indicators of functionally active or inactive state.

### Upstream

**Figure 3:**
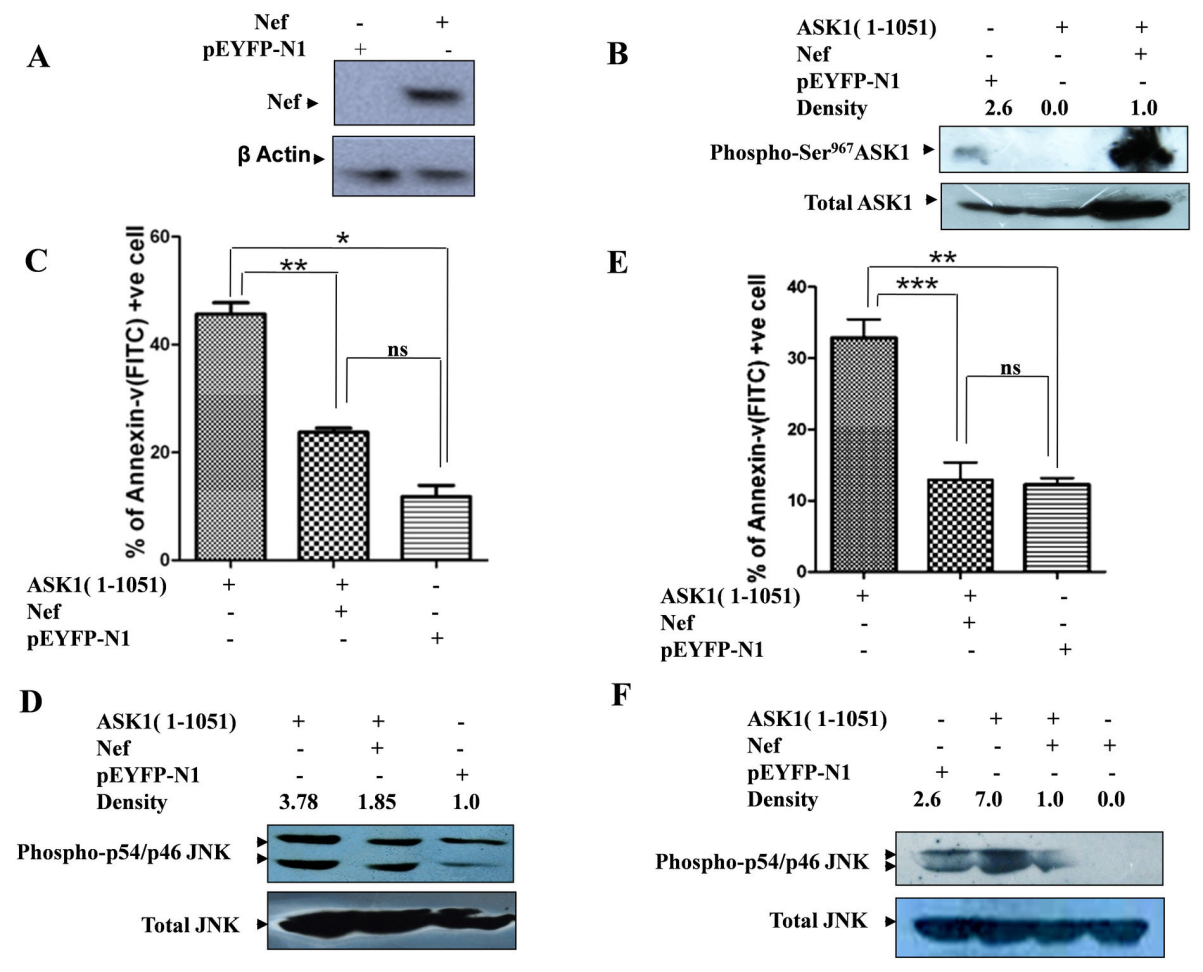
Inhibition of ASK1 (1-1051 aa) induced apoptosis by Nef. (**A**) **Expression of Nef:** HEK-293 cell was transfected with pEYFP-N1-Nef plasmid and vector back bone. Cells were harvested after 48 hrs and western bolting was done by anti Nef antibody. Showing stable expression of Nef protein. (**B**) HEK-293 cell was transfected with ASK1(1-1051 aa) along with/without Nef showing ASK1-Ser^967^ phosphorylation. ASK1-Ser^967^ was very low in vector control and ASK1 transfected HEK-293 cells, co-transfection with Nef significantly potentiated ASK1-Ser^967^ phosphorylation. (**C**) HEK-293 cells transfected with ASK1 (1-1051 aa) exhibited apoptotic cell count of 45.65% while HEK-293 cell that were co-transfected with ASK1 (1-1051 aa) and Nef (full length) exhibited 23.61% apoptotic cell count. pYFP-N1 vector control exhibited an apoptotic cell count of 11.78%. Cells that harbored vector backbone served as control. The data are means +SEM from three distinct experiments. *P* values of <0.05 were obtained from two-tailed paired *t* test. (**D**) p54/46JNK1 pathway activation was studied using western blot /densitometry analysis of HEK-293 cells that were transfected with ASK1 (1-1051 aa) with/without Nef. (**E**) Annexin-V/PI labeling followed by flow cytometric analysis of ASK1(1-1051 aa) transfected Jurkat T cell revealed 32.85% Annexin-V positive apoptotic cell count, While Jurkat T cell that were co-transfected with ASK1(1-1051 aa) and Nef (full length) exhibited 12.9% apoptotic cell count. Vector control (pYFP-N1) showed 12.79% Annexin-V positive apoptotic cells. (**F**) Jurkat T cell were transfected with plasmid encoding ASK1 (1-1051 aa) with and without plasmid encoding HIV-1Nef. Cells were harvested after 40 hrs of transfection and western blot analysis for JNK1 activation was done using anti phospho-p54/46JNK1 and p54/46JNK1 antibodies showing relative levels of activated p54/46JNK1 i.e. ratio between integrated density of phospho-p54/46JNK1 and p54/46JNK1 bands. All western bolting was done three times and integrated density was determined by densitometry analysis using imageJ software.

ASK1 has two important upstream negative regulators viz. Thioredoxin and 14-3-3 protein (Negative regulator). It has been reported that under basal conditions ASK1 amasses with thioredoxin, a cellular factor that inhibits its catalytic function. The inhibitory action of thioredoxin on ASK-1 is affected through redox reaction at critical ASK1-Cys^250^ residue [27]. Unlike the well delineated modulation of ASK1-thioredoxin interaction by Nef, the effect of Nef on ASK1-14-3-3 interaction remains largely obscure. Our observation that Nef interacting C-terminal ASK1 region (905-1051 aa), harbored a critical 14-3-3 binding Ser^967^ residue points towards the possibility of Nef being able to modulate ASK1-14-3-3 interaction. In order to address this issue we evaluated the effect of Nef on ASK1-Ser^967^ phosphorylation. ASK1-Ser^967^ being a critical component of 14-3-3 interaction site serves as crucial integration node that couples survival signaling pathways to this death promoting kinase [28]. Carefully orchestrated cycles of phosphorylation/dephosphorylations at ASK1-Ser^967^ by upstream kinases/phosphatases regulate binding of ASK1 to its negative regulator viz. 14-3-3 protein [29]. We cloned different ASK1 fragments and Nef (full length) and Nef core domain (57-207) in mammalian expression vector pEYFY-N1 vector which makes recombinant protein with YFP protein. Figure 3A represents western blot analysis showing stable expression of HIV-1Nef protein.

ASK1 fragment was transfected in HEK-293 and/or Jurkat T cell with and without Nef. After 48 hrs of transfection cells were harvested for evaluating ASK1-Ser^967^ phosphorylation and apoptosis using western blot analysis and Annexin-V/PI labeling assay respectively. While ASK1 was largely present in functionally inactive state (phosphorylated at Ser^967^) in cells harboring vector backbone, as evident by elevated p-Ser^967^ASK1 levels, the minimal ASK1 transfected HEK-293 largely contained functionally active form of ASK1 as indicated by much diminished ASK1-Ser^967^ phosphorylation. Interestingly, co-transfection with Nef potentiated ASK1-Ser^967^ phosphorylation considerably. Phosphorylation of this residue is known to enable ASK1 to bind to 14-3-3 leading to suppression ASK1 induced apoptosis (Figure 3B). Consequently enhanced ASK1-Ser^967^ phosphorylation may be one of the key means employed by Nef to modulate ASK1-14-3-3 interaction and thereby impair ASK1 pro-apoptotic function. In agreement with this HEK-293 and Jurkat T cells co-transfected with ASK1(1-1051 aa) and Nef exhibited a much diminished apoptotic count viz. 23.18% and 12.97% respectively as against ASK1 (1-1051 aa) transfected HEK-293 cells and Jurkat T cells which exhibited an apoptotic cell count of 44.45% and 32.85% respectively (Figure 3C & 3E). Findings not only established the pro-apoptotic function of truncated ASK1 (1-1051 aa) but also demonstrated marked inhibition of ASK1 (1-1051 aa) induced apoptosis by Nef at least in part by modulation of ASK1-14-3-3 interaction.

### Downstream

Full length ASK-1 MAPKKK has been proposed to induce apoptosis through phosphorylation and consequent activation of downstream effectors cascades viz. JNK1 and/or p38 pathway. In view of this we looked for activation of JNK1 and p38 pathways in HEK-293 cells that were transfected with ASK1 (1-1051 aa) either in presence or absence of Nef. Western blot analysis revealed activation of JNK1 and p38 pathways in ASK1 transfected HEK-293 cells, albeit at different time points. While JNK1 activation occurred 40 hrs post transfection, p38 pathway remained largely unaffected at this time point and exhibited activation much later i.e. 48 hrs post transfection (Figure S1). In view of JNK1 activation being an earlier event, it was employed as a marker of ASK1 function in subsequent studies. In concurrence with above, ASK1 transfected in HEK-293 cells and Jurkat T cells also exhibited JNK1 activation as indicated by elevated phosphorylation JNK1 levels (Figure 3D & 3F). Interestingly, the observed ASK1 dependent activation of JNK1 was markedly impaired when cells were co-transfected with Nef. Thus we demonstrated that Nef interacted with and thereby inhibited pro-apoptotic function of ASK1 (1-1051 aa). We also transfected HEK-293 cells with ASK1 fragment (319-1051 aa), ASK1 (1-904 aa), and ASK1 (319-904 aa) with and without Nef. We observed that inhibitory effect of Nef on ASK1 induced apoptosis was more prominent when ASK1 contain at both N- terminal (1-318 aa) and C- terminal (905-1051 aa) Nef interacting regions compared to ASK1(319-1051 aa) and ASK1(1-904 aa) alone. As expected the inhibitory effect of Nef was not evident when cells were transfected with ASK1 (319-904 aa), a region that didn’t interact with Nef at all (Figure S2). It has been reported that over expression of wild-type ASK1 causes induction of apoptosis by JNK-1 and p38 phosphorylation [30]. Here we have showed for the first time that truncated ASK1 (1-1051 aa) is also able to induce apoptosis and that it contains two minimal essential domain (1-318 aa and 905-1051 aa) through which Nef interacts with ASK1 (1-1051 aa) and subsequently inhibits its pro-apoptotic function.

### Competitive binding of peptide to disrupt HIV-1Nef-ASK-1 Interaction

**Figure 4:**
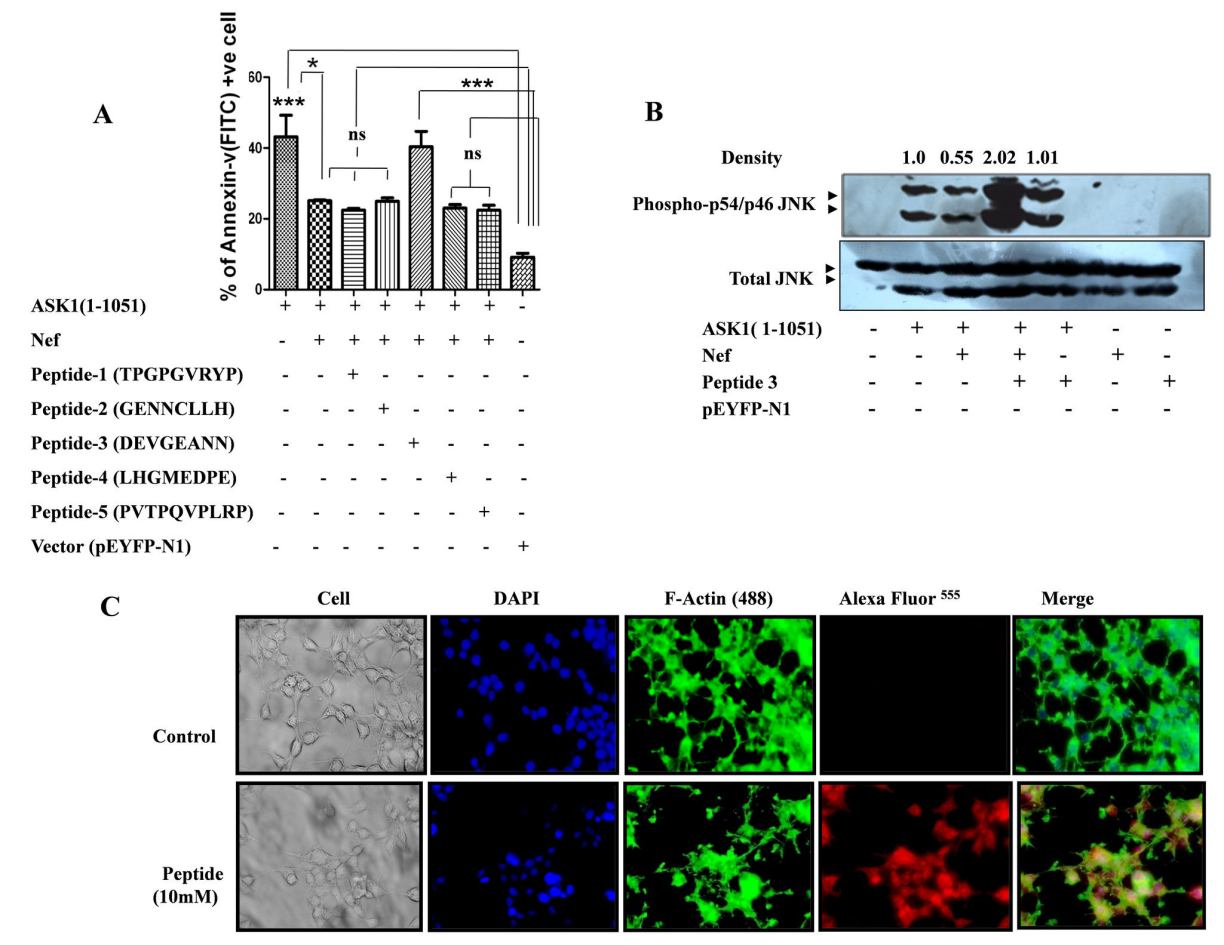
Compatative binding of peptide to modulate ASK1-Nef mediated anti-apoptotic function. (**A**) HEK-293 cells that were transfected with ASK1 (1-1051 aa) in absence or presence of Nef exhibited Annexin-V positive apoptotic cell count of 43.15% and 25.11% respectively. Treatment of ASK1 (1-1051 aa) and HIV-1Nef co-transfected with 10 µM of peptide-1, peptide-2, peptide-3, peptide-4 and peptide-5 after 24 hrs of transfection resulted apoptotic cell count of 22.45%, 24.96%, 40.37%, 23.0% and 22.45% respectively. Cells that harbored vector backbone served as control. The data are means +SEM from three distinct experiments. *P* values of <0.05 were obtained from two-tailed paired *t* test. (**B**) Effect of peptide- 3 was evaluated on ASK1 mediated p54/46JNK1 pathway activation. HEK-293 cells harboring plasmids encoding ASK1 (1-1051 aa) and HIV1Nef were treated with 10 µM of peptide-3 (152DEVGEANN159), precisely 24 hrs post transfection. Cell were harvested after 40 hrs followed by western blot/densitometry analysis to determine relative extent of activation (ratio between integrated density of phospho-p54/46JNK1 and p54/46JNK1 levels). The ability of HIV-1Nef to impair ASK1 (1-1051 aa) function i.e. p54/46JNK1 phosphorylation markedly impaired in presence of peptide-3. (**C**) **Peptide internalization**: 10 mM peptide Alexa flour^555^ labeled peptide was added 1×10^5^/well cell seeded on glass cover slip in 24 well plates and was incubated for 24 hrs in HEK-293 cell and cell were stain with DAPI. Image was taken at red filter and blue and pictures were merged.

Truncation of 66 aa from C-terminal of Nef significantly impaired its interaction with ASK1 (Figure 2). This gave an indication that the region might contain potential sites of interaction with ASK1 (1-1051 aa). Prompted by this we synthesized the various peptides of sequences that were identical to C-terminal amino acid sequences of Nef protein and thus might compete for interaction with ASK1 (1-1051 aa). It has been reported that 73 PXXPXR78 domain interacted with MAPK (Hck) and this domain also interacts with Src family kinase [31]. Therefore we also took this domain in one particular peptide sequence (Peptide-5) so as to decipher if this motif interacts with ASK1. HEK-293 cells were co-transfected with ASK1 (1-1051 aa) and Nef (Full length) followed by addition of 10µM of peptide/vehicle precisely 24 hours after transfection. HEK-293 cells co-transfected with ASK1(1-1051 aa) and full length Nef underwent apoptosis to a much lesser extent (25.1%) as compared to cells transfected with ASK-1 alone (43.15%). Post transfection addition of various peptides viz. Peptide-1, Peptide-2, Peptide-3, peptide-4 and Peptide-5 accounted for 22.45%, 24.96%, 40.37%, 23.0% and 22.45% apoptotic cells respectively (Figure 4A). As evident, the peptide-3 sequence N-DEVGEANN-C restored the commencement of apoptosis to a level almost comparable to that in ASK-1 transfected cells, thus it considerably modulated the Nef mediated anti-apoptotic function. This result suggested that peptide sequence N-DEVGEANN-C competitively inhibited interaction of ASK1 with 152DEVGEANN159 motif of Nef.

To validate our findings that Nef-ASK1 interaction mediated apoptosis could be reversed by peptide, we treated ASK1-Nef co-transfected HEK-293 cells with 10µM peptide-3. In concurrence with our earlier observation wherein peptide-3 significantly reversed anti-apoptotic effect of Nef in ASK1 transfected cells, here again the peptide-3 treatment 24 hrs post transfection significantly potentiated JNK1 activation. In agreement with this, the localization studies using Alexafluor^555^ labeled peptide 3, revealed significant internalization of peptide-3 within HEK-293 cells (Figure 4C). This result substantially corroborated that peptide (152DEVGEANN159) contained essential residues which enabled it to successfully compete with HIV-1Nef for interaction with ASK1 (1-1051 aa) and thereby circumvent the anti-apoptotic effect of Nef, leading to restoration of ASK1 pro-apoptotic functions.

### Mutation in Nef 152DEVGEANN159 domain results in loss of interaction with minimal ASK1 and Anti-apoptotic function:

**Figure 5:**
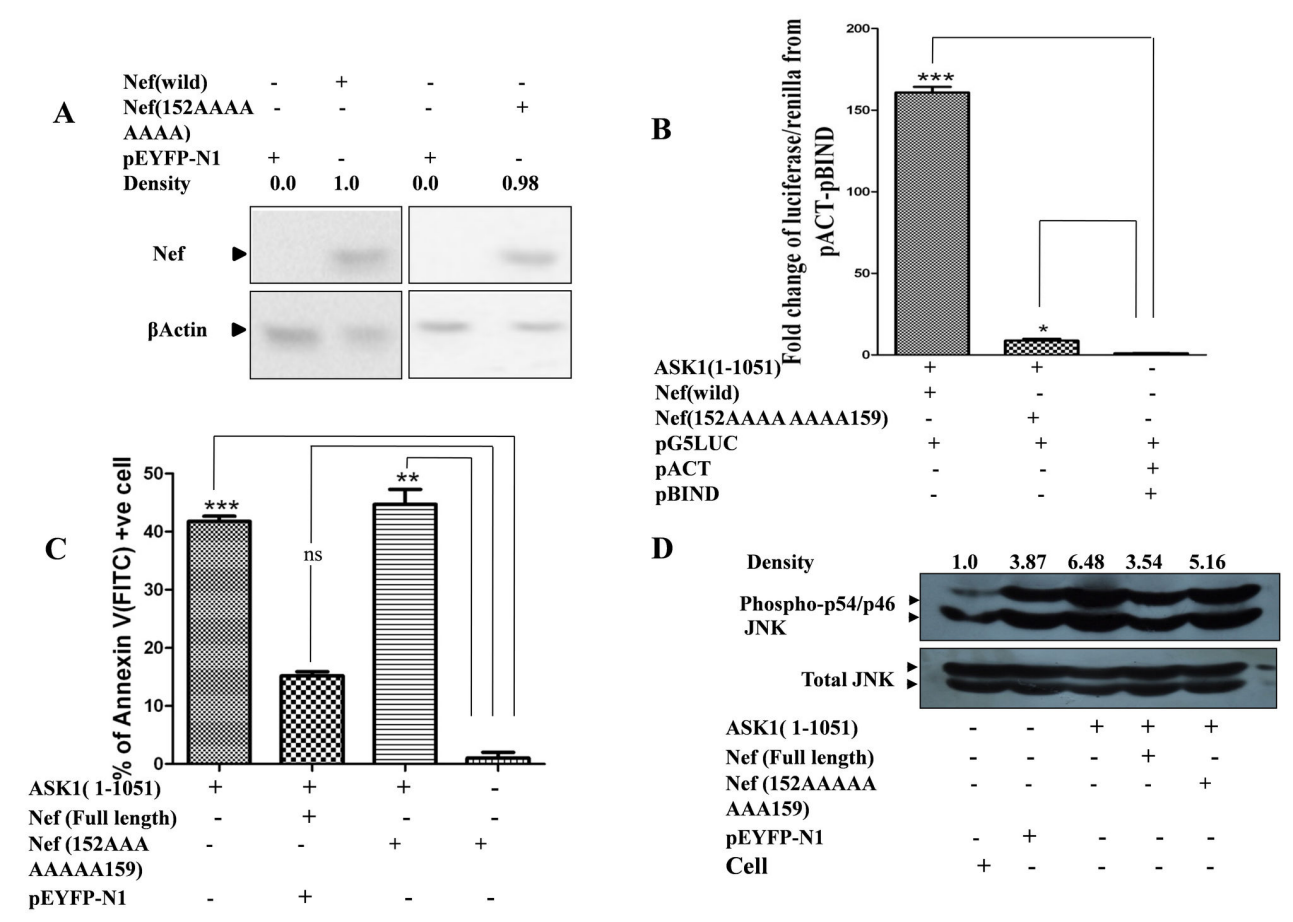
Mutation with in 152DEVGEANN159 region of Nef impairs its ability to interact with and restore ASK1 functions. (**A**) HEK-293 cell was transfected with pYFP-N1Nef (152AAAAAAAA159) plasmid and vector back bone. Cells were harvested after 48 hrs and western blotting was done by anti-Nef antibody. Showing almost equal level of protein expression. (**B**) Mammalian two hybrid assay was done for ASK1 (1-1051 aa), Nef and mutated HIV-1Nef (152-AAAAA159). Plasmid encoding GAL4-ASK1 was cloned in pBIND vector and Vp-16-Nef was cloned in pACT vector. HEK-293 cells co-transfected with ASK1 (1-1051 aa), Nef and pG5LUC vector exhibited a 156 fold increase in luciferase/renilla expression as compared to pACT-pBIND vector control. GAL4-ASK1 (1-1051 aa) and Nef (152AAAAA159) co-transfected cell in combination with pG5LUC showed only 8.75 fold increase in luciferase/renilla expression over from pACT-pBIND. The data are means +SEM from three distinct experiments. *P* values of <0.05 were obtained from two-tailed paired *t* test. (**C**) Effect of mutated HIV-1Nef (152-AAAAA159) on ASK1 (1-1051 aa) mediated apoptosis was evaluated using flow cytometric analysis of Annexin-V/PI labeling assay. Mutation with in critical regions of HIV-1Nef markedly affected anti-apoptotic potential of Nef as indicated by much elevated apoptotic cell count viz. 44.6% in case of HIV-1Nef (152-AAAAA159) as opposed to 15.16% in case of Nef. The data are means +SEM from three distinct experiments. *P* values of <0.05 were obtained from two-tailed paired *t* test. (**D**) Effect of mutated HIV-1Nef (152-AAAAA159) on ASK1 (1-1051 aa) mediated p54/46JNK 1pathway activation was studied using western blot /densitometry analysis of HEK-293 cells that were transfected with ASK1 (1-1051 aa) with/without Nef or mutated HIV-1Nef (152-AAAAA159). Mutation with in critical regions of HIV-1Nef altered p54/46JNK1 activation profile markedly as indicated by higher phospho-p54/46JNK1 levels. Western blotting was done three times showing consistent result.

To further substantiate that indeed the corresponding N-DEVGEANN-C within Nef interacted with ASK1 leading to inhibition of ASK1 mediated apoptosis, the amino acids with 152DEVGEANN159 domain of Nef were replaced with alanine to obtain a mutated Nef (152AAAAAAAA159). Western blot studies (Figure 5A) revealed that this alteration did not impair the expression of protein product and level of expression of mutant Nef were comparable to that of wild type Nef.

Analysis of interaction affinity using mammalian two hybrid assay revealed that replacement of 152 DEVGEANN159 domain with 152AAAAAAAA159 impaired the ability of Nef to interact with ASK1(1-1051 aa) as indicated by diminished luciferase/renilla expression (Figure 5B). Our next objective was to investigate the effect of mutation in Nef on ASK1 (1-1051 aa) induce apoptosis. We found that HEK-293 cells co-transfected with ASK1 and the mutated Nef (152AAAAAAAA159) underwent apoptosis to a much greater extent than the wild type Nef (N-DEVGEANN-C) (Figure 5C). Furthermore the JNK1 pathway was also hyper activated in cells co-transfected with mutated Nef (152AAAAAAAA159) as compared to wild type Nef (N-DEVGEANN-C) (Figure 5D). The results substantiated the importance of constituent N-DEVGEANN-C domain for Nef to be able to interact with and thereby impair the minimal ASK1 regulated downstream signaling.

### Molecular mechanisms up and downstream to Endogenous ASK1: Deregulation due to Nef - Endogenous ASK1 interaction and its reversal by synthetic peptide:

**Figure 6:**
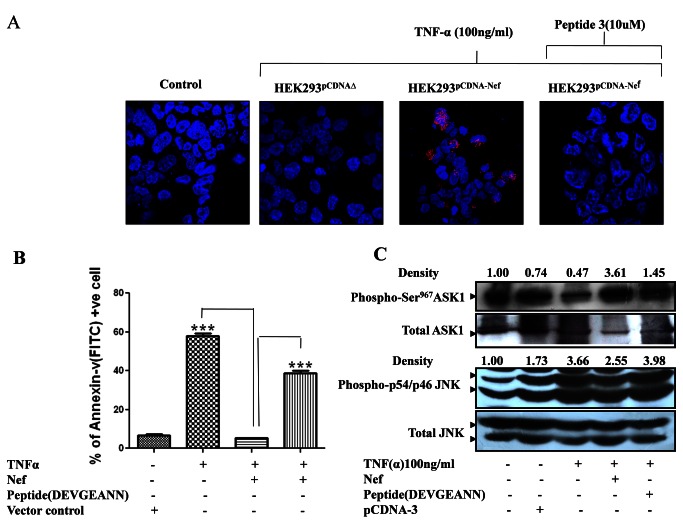
Synthetic peptide N-DEVGEANN-C successfully inhibited Nef from interacting with and there by restore the functions of endogenous wild type ASK1 activity. (**A**) HEK-293 cells were transfected with Nef plasmid or vector backbone. 24 hrs post transfection, cells were stimulated to progress through ASK1 mediated apoptotic progression using classic ligand viz. TNF-α (100ng/ml) either in absence or presence of synthetic peptide N-DEVGEANN-C (10ng/ml). The extent of interaction of endogenous wild type ASK1 with Nef and ability of peptide N-DEVGEANN-C to impair the interaction was evaluated using *In situ* proximity ligation assay. (**B**) Synthetic peptide N-DEVGEANN-C prevented HIV-1Nef from offering protection against TNF -α induced, endogenous wild type ASK1 mediated apoptosis as evaluated using Annexin-V/PI assay. (**C**) Endogenous wild type ASK1 activity was rendered active by synthetic peptide N-DEVGEANN-C even in presence of HIV-1Nef as indicated by markedly decreased inhibitory Ser^967^ phosphorylation of ASK1 with a corresponding increase in p54/46JNK1 activation profile as indicated by higher phospho-p54/46JNK1 levels in western blot analysis. All western blotting was done three times and integrated density was determined by densitometry analysis using imageJ software.

Having studied the physical interaction of Nef with minimal ASK1 (1-1051 aa) we next decided to extend our studies to interaction of Nef with wild type endogenous ASK1. For studying interaction of Nef with wild type endogenous ASK1, the death receptor signaling with in vector/Nef transfected HEK-293 cells was stimulated using TNF-α followed by *in situ* proximity ligation assay. Cells where proximity probes will gain close proximity due to physical interaction between protein (Nef and endogenous ASK1) pair were expected to exhibit fluorescent foci due to localized accumulation of RCA product. As expected these foci were completely absent in control (unstimulated cells) and vector control cells (stimulated with TNF-α) indicating absence of physical interaction. On the other hand Nef exhibited considerable physical interaction with wild type endogenous ASK1 as evident by presence of numerous fluorescent foci in TNF-α stimulated HEK-293 cells that harbored Nef (Figure 6A). In agreement with this, stimulation with TNF-α while markedly potentiated apoptosis in control cells, it failed to elicit similar response in cells harboring Nef (Figure 6B). The effects of Nef on functional integrity of wild type endogenous ASK1 was further evaluated by measuring the extent of inhibitory Ser^967^ phosphorylation and phosphorylation of its downstream target i.e. JNK1 following stimulation of HEK-293 cell with TNF-α. Ability of Nef to compromise functional integrity of wild type endogenous ASK1 became evident when, compared to Nef deficient HEK-293 cells, the Nef expressing HEK-293 cells exhibited enhanced p-Ser^967^ ASK1 levels and diminished p-54/46 JNK1 levels in response to stimulation by TNF-α (Figure 6C).

**Figure 7:**
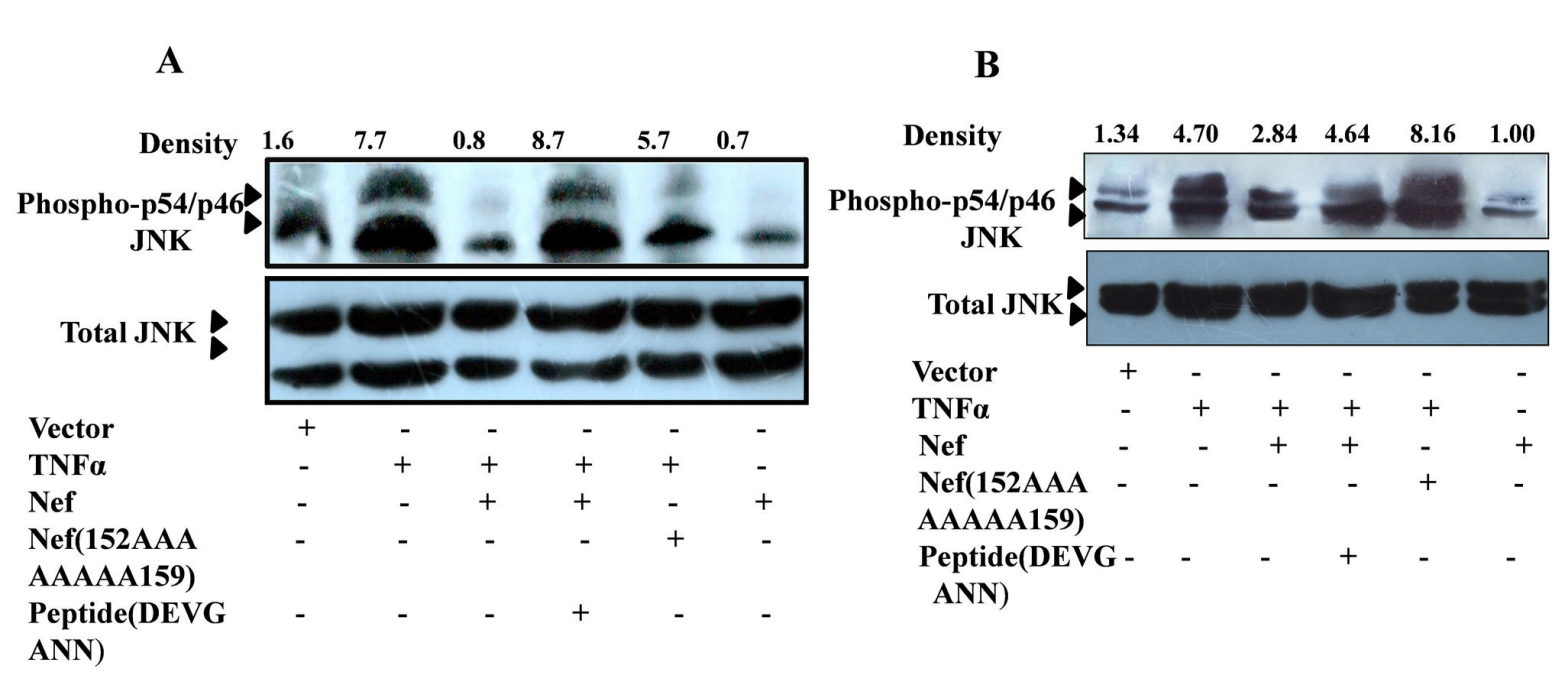
Synthetic peptide N-DEVGEANN-C and Nef(152AAAAAAAA159) restore endogenous ASK1 activity in SupT1 and Jurkat T cell. (**A**) Jurkat T cells were transfected with Nef plasmid, Nef (152AAAAAAAA159) plasmid or vector backbone. 24 hrs post transfection, cells were stimulated to progress through ASK1 mediated apoptotic progression using usual ligand viz. TNF-α (100ng/ml) either in absence or presence of synthetic peptide N-DEVGEANN-C (10ng/ml). Cells treated with TNF-α showing higher phospho-p54/46JNK1 levels and Nef, Nef (152AAAAAAAA159) transfected cell show p54/46JNK1 activation profile as indicated by in western blot analysis lower and high respectively. (**B**) SupT1 cells were transfected with Nef plasmid, Nef (152AAAAAAAA159) plasmid or vector backbone. 24 hrs post transfection, cells were stimulated to progress through ASK1 mediated apoptotic progression using usual ligand viz. TNF-α (100ng/ml) either in absence or presence of synthetic peptide N-DEVGEANN-C (10ng/ml). Cells treated with TNF-α showing higher phospho-p54/46JNK1 levels and Nef, Nef (152AAAAAAAA159) transfected cell show p54/46JNK1 activation profile as indicated by in western blot analysis lower and high respectively.

Having established the inhibitory potential of peptide (N-DEVGEANN-C) against interaction of Nef with minimal ASK1 (1-1051 aa), we also explored the efficacy of the peptide inhibitor to impair the interaction of Nef with wild type endogenous ASK1 in HEK-293 cells. Using in situ proximity ligation assay in control and TNF-α stimulated, cells we successfully demonstrated that peptide (N-DEVGEANN-C) hindered interaction of Nef with wild type endogenous ASK1 (Figure 6A). We also evaluated the effect of peptide inhibitor on Nef-ASK1 interaction in terms of functional integrity of wild type ASK1. Interestingly the peptide (N-DEVGEANN-C) inhibitor restored TNF-α induced ASK1 activation even in presence of Nef as indicated by markedly increased apoptotic cell count and diminished levels of functionally inactive form of ASK1 i.e. p-Ser^967^ASK1 (Figure 6B & 6C). In agreement with this, the wild type endogenous ASK1 activity within TNF-α stimulated, Nef transfected cells was markedly potentiated in presence of peptide (N-DEVGEANN-C) as indicated by enhanced phosphorylation of its downstream substrate viz. JNK1(Figure 6C). Our results established that similar to minimal ASK1, the interaction of Nef with wild type endogenous ASK1 is also through N-DEVGEANN-C domain and that is why the identical synthetic peptide (N-DEVGEANN-C) could not only successfully compete with Nef for interaction with wild type ASK1 but was able to reverse inhibitory effects of Nef. Since T cell subpopulation remains the primary target during HIV-1 infection, the findings obtained in HEK-293 cells were further corroborated in Jurkat T cells and Sup T1 cells. Results revealed that identical synthetic peptide (N-DEVGEANN-C) successfully competed with Nef for interaction with wild type ASK1 in these cells lines as well, thereby substantiating the involvement of 152 DEVGEANN159 in ASK1-Nef interaction (Figure 7).

## Discussion

The host immune defense mechanism is programmed to selectively eliminate HIV-1 infected cells. The viral protein HIV-1Nef confers survival advantage to infected cells by stalling death receptor signaling. Consequent delay of the apoptosis culminates in the synthesis of new virions.

The inhibition of apoptosis in infected cells is caused by the physical interaction of HIV-1 Nef with ASK1. However, the ASK1 and Nef domains directly participating in the interaction were not known. Using mutation studies, efforts were made to identify specific domain through which Nef might inhibit TNF-α mediated apoptosis in Jurkat T cells [8]. Employing truncated variants of ASK1, in the current study, we have identified regions on the ASK1, which were vital for its interaction with Nef protein. Interestingly, the identified regions viz 1-345 aa and 861-1051 aa of ASK1 harbored binding sites for regulatory proteins TRX and 14-3-3 respectively (Figure 1A). Both regions individually exhibited propensity to bind with Nef albeit with different affinity.

The obvious question was how would ASK1 variant (1-345 aa and 861-1051 aa) housing both the interacting regions would interact with Nef. Our results clearly demonstrate that the affinity of ASK1 (1-1051 aa) was substantially higher than variant housing either of the regions. The increased affinity of the ASK1 variant (1-1051 aa) demonstrated that this region was sufficient for interaction with Nef (Figure 1B). Thus we designated this region of ASK1 as minimal ASK1 region since it contain two critical domains (1-318 aa & 905-1051 aa Figure 1B) which was important for its interaction with Nef.

We also carried out studies to identify the region of Nef that interacted with minimal ASK1. HIV-1 Nef has three major regions viz N terminal unstructured myristoylation region (1-72 aa), conserved region (73-148 aa and 179-207 aa) and C terminal (149-178 aa) which is again unstructured region [32---]. Deletion variants of Nef showed varied interaction affinity towards minimal ASK1. The Nef variant lacking unstructured C-terminal region (140-207 aa) exhibited reduced affinity towards minimal ASK1 (Figure 2A and Figure 2B). This strategy led to identification of Nef regions that potentially interacted with minimal ASK1.

At cellular level, the consequence of Nef-ASK1 interaction is impairment of ASK1 mediated death receptor signaling. Over expression of ASK1 induces apoptosis in HEK-293 and Jurkat T cells [33]. In agreement with this our studies on minimal ASK1 showed that expression of minimal ASK1 induces apoptosis in HEK-293 and Jurkat T cells (Figure 3B & 3D). Co-expression of Nef with minimal ASK1 inhibits the minimal ASK1 induced apoptosis. Furthermore the upstream and downstream signaling pathways initiated by minimal ASK1 to culminate into apoptosis were also inhibited by Nef.

Presence of Nef inhibits ASK1 catalytic activity by impairing simultaneous coupled release of its normal negative regulator thioredoxin [8]. In our studies affinity of minimal ASK1, towards the Nef increased substantially in presence of C terminal region (905-1051 aa) of ASK1. This region contained binding site for another regulatory protein via 14-3-3, a negative regulator of ASK1 [34].

The catalytically inactive ASK1 remains phosphorylated on serine^967^ while dephosphorylated ASK1 is catalytically active [28]. 14-3-3 protein exercises regulatory control over ASK1 through interacting with phosphorylated ASK1 Ser^967^. Interestingly presence of Nef markedly potentiated ASK1-Ser^967^ phosphorylation, an event that enables ASK1 to bind 14-3-3 protein, leading to suppression of ASK1 induced apoptosis. In view of ASK1-Ser^967^ phosphorylation being a critical prerequisite for its association with 14-3-3, the ability of Nef to potentiate p-Ser^967^ASK1 might affect sustained association of 14-3-3 with ASK1 leading to impaired ability of ASK1 to activate downstream effectors of apoptosis such as JNK1 and p38.

The mechanism as to how the HIV-1 Nef would modulate Serine^967^ phosphorylation remains to be explored.

ASK1 executes apoptosis by phosphorylation and activating JNK1 and p38 kinase [20]. Our minimal ASK1 also phosphorylated JNK1 and p38. The phosphorylation of JNK1 was reversed when Nef was co-expressed with minimal ASK1. In our studies, minimal ASK1 could delineate the importance of both regulatory regions on the ASK1 for its interaction with Nef and this interaction could reverse the upstream and downstream signaling cascades in co-transfected cells (minimal ASK1 and Nef). This interaction impaired pro-apoptotic functions of ASK1 (Figure 3C & 3E).

Although minimal ASK1 (1-1051 aa) harbors both regulatory regions, yet it lacked 1052-1374 aa region which is present in endogenous ASK1. The death receptor dependent activation of ASK1 involves dissociation of TRX from N terminal and dephosphorylation of serine^967^ residue followed by activation of downstream effectors viz JNK1/p38 with a final culmination into apoptosis. Using TNF-α as death receptor stimulator, we could demonstrate that similar to minimal ASK1, the endogenous ASK1 also remained inhibited in presence of Nef. Nef expression in HEK-293 cells, markedly potentiated serine^967^ phosphorylation of ASK1, and inhibited JNK1/P38 phosphorylation despite TNF-α stimulation (Figure 6C). Accordingly apoptosis also remained inhibited.

We recently reported crystal structure of tetrameric Nef complex wherein the above mentioned 141-207 aa region appeared in disordered form [35]. Ability of dynamic loops to regulate different protein by way of conformation dependent adequate positioning is well documented. Consistent with this, the motif was found to contain di-Leu and EE motifs as well as acidic residue clusters that are known to interact with different sets of host accessory proteins [36]. Therefore we hypothesized that peptide having a sequence identical to ASK1 interacting domain will compete with Nef for interaction. Several peptide octamers were designed spanning C terminal unstructured region (129-178 aa) of Nef. Amongst all, the peptide-3 (N-DEVGEANN-C) successfully competed with Nef for interaction with both endogenous and minimal ASK1 (Figure 4A, 4B and Figure 6A) and reversed inhibitory effects of Nef on TNF-α-ASK1-JNK1 axis. Based on this, the corresponding region in Nef was mutated. The mutated Nef could neither interact with minimal ASK1 (Figure 5A) nor limit the anti-apoptotic function of HIV-1Nef (Figure 5B & 5C). These results confirmed that (152-DEVGEANN-159) sequence on C terminal region of Nef is the novel motif interacting with ASK1. Thus our study led to identification of constituent domains of both Nef and ASK1 that are essential for physical interaction between Nef and ASK1.

## Materials and Methods

### Antibodies and Reagents

Anti-p-Ser^967^ASK1, anti-ASK1 and anti-HIV-1Nef antibodies were purchased from Abcam (USA). Anti-p-JNK, anti-JNK1 antibodies were purchased from Cell Signaling Technology (USA). HRP conjugated goat anti-rabbit IgG was purchased from Jackson Immuno Research Europe Ltd. (U.K). MEM, RPMI1640 and antibiotics were purchased from Sigma, USA. Transfection reagent ExGen 500 and restriction enzymes were purchased from Fermentas (USA). Endotoxin free plasmid isolation kit, Two-Hybrid System of protein–protein interaction, was purchased from Promega (USA). Rink amide MBHA resin (loading capacity: 0.4–0.8 mmol/g) and all the N-α Fmoc and necessary side chain protected amino acids were purchased from Novabiochem, Switzerland. Coupling reagents for peptide synthesis like 1-hydroxybenzotriazole (HOBT), di-isopropylcarbodiimide (DIC), 1, 1, 3, 3-tetramethyluronium tetrafluoroborate (TBTU), and N,N′-diisopropylethylamine (DIPEA) were purchased from Sigma, USA. Dichloromethane, N,N′-dimethylformamide (DMF), and piperidine were of standard grades and procured from reputed local companies. Acetonitrile (HPLC grade) was procured from Merck, India. Trifluoroacetic acid (TFA) and trifluoroethanol (TFE) were purchased from Sigma. DuoLink proximity ligation assay (PLA) kit was purchased from OLink Biosciences (Sweden).

### Cell Culture and Transfection

Jurkat T cell, Sup T1 cells were purchased from ATCC and HEK-293 cell culture was procured from Tissue and Cell Culture Unit, Central Drug Research Institute, India. HEK-293 cells and Jurkat T cells, Sup T1 cells were maintained in MEM and RPMI-1640 respectively. Growth media was supplemented with 10% FBS (invitrogen), 100µg/ml penicillin, 100µg/ml streptomycin and 2.5ng/ml amphotericin B. Cells were grown using standard cell culture techniques in humidified atmosphere (95% humidity) at 37°C and 5% CO_2_. Transient transfection experiments were carried out using log phase HEK-293 cells. Briefly, 1×10^5^ HEK-293 cells were allowed to enter log phase by maintaining them overnight in complete growth media. A 3µg endotoxin free plasmid preparation was mixed with 10 µl of ExGen 500 and volume was made up to 150 µl by 1×PBS. Reaction mixture was vortex and kept for 10 minute at room temperature. After 10 minute reaction mixture was overlay on HEK-293 followed by addition of 1ml complete MEM. Thereafter cells were maintained in humidified atmosphere (95% humidity) at 37°C and 5% CO_2_. Transfection in Jurkat T cells was done by electroporation. Briefly, 4×10^6^ cell were seeded in 10 ml RPMI-1640 media. After 24 hr cell were harvested at 405g and resuspended in 400µl incomplete RPMI (containing no FBS and antibiotics). A 20 µg endotoxin free plasmid preparation was introduced to cell suspension followed by electroporation with gene pulser (BTX) (250 V, 950mF) incorporating a green Fluorescent protein (GFP) expression vector to mark the transfected cells. After electroporation cell were grown in incomplete RPMI (Serum free) for 2hr and thereafter in complete media containing 20% FBS.

### Construction of Plasmid for Mammalian Two Hybrid System of Protein-Protein Interaction

ASK1 clone of accession no. BC054503, originally purchased from Saif lab was cloned in pCMV SPORT plasmid. To identify which particular motifs in ASK1 are required for interaction with Nef, a series of truncation with overlapping gene construction were made (Figure 1A). ASK1 fragments were amplified by primers whose sequences are given in table 1. These overlapping fragments were cloned in pBIND vector of mammalian two hybrid system which generated recombinant protein with GAL4 binding. Nef gene (accession No GQ184335) was cloned in pACT vector in frame of activation domain of pACT vector. Simple examination of full length Nef cloned in pACT vector might not have allowed us to detect the interaction between Nef and ASK1 as its anchor domain upon myristoylation [37,38] might keep it localized to plasma membrane thereby rendering it incapable to enter nucleus. To eliminate this false negative outcome, the mammalian two hybrid studies were conducted using a truncated Nef that was devoid of anchor domain (1-57 aa) and contained only core domain (57-207 aa). Rest of the studies was carried out using the biologically active full length Nef.

**Table 1:** 

**Primer for ASK1 cloned pBIND**.	
F1	5' GGATCCGTATGAGCACGGAGGCGG 3'
R345	5' TCTAGAGCCAAATCAAAGGTTGGCAGTTT 3'
F319	5' GGATCCGTCTTTCCTACAGAGATATCCA 3
R670	5' TCTAGACAAGTCACTTTCACAGTCTCCTTC 3'
F607	5' GGATCCGTTCTTCTGTCAGGGGAGT 3'
R904	5' TCTAGATGGGATCTCAGGGTGGACTTTA 3'
F861	5' GGATCCGTGCAGCAGACATCTGGTCT 3'
R1051	5' TCTAGATCGCCTCTCACTGTCCTTCCTC 3'
F1059	5' GGATCCGTACGGAAGACCAAGACAAAATT 3'
R1182	5' TCAGATGCAAGGCTGAAATGTGGCCTTA 3'
F1152	5' GGATCCGTATGTTTGCCTTAGACAGTATCA 3'
R1374	5' TCAAGTCTGTTTGTTTCGAAAGTCAATGA 3'

### Creating ASK1 Truncations and Different Nef constructs:

Several truncations of viz. ASK1 (1-1051 aa), ASK1 (1-904 aa), ASK1 (319-1051 aa) and ASK1 (319-904 aa) and different Nef construct were cloned in pEYFP-N1 in pEYFP-N1 (Novagen) vector for which the primer sequence are given in table (2).

**Table 2:** 

Primer	sequence 5’----------------------------------------------3’	
**Primer for ASK1 cloned in pEYFP-N1**		
F1(YFP)	5'GTCGACATGAGCACGGAGGCGGACGA3'	
F319(YFP)	5' GTCGACATGCTTTCCTACAGAGATATCCAGGA 3'	
R904(YFP)	5' GGATCCGCTGGGATCTCAGGGTGGA 3'	
R1051(YFP)	5' GGATCCACTCGCCTCTCACTGTCCTTCCTCA 3'	
**Primer for Nef cloned in pEYFP-N1**		
NeFFP(YFP)	5'AGCAAGCTTATGGGGGGCAAGTGGTCAAAA3'	
NeFRP(YFP)	5'AGCGTCGACGCGCAGTCTTTGTAATACTCCG3'	
**Primer (for Nef mutation)**		
NefFP (1-454)		5'AAGCTTATGGGGGGCAAGTGGTCAAA3'
Nef mutFP		5'GCTGCAGCAGCAGCAGCCGCTGGAGAGAA3'
Nef mut RP		5'AGCGGCGGCTGCTGCTGCTGCAGCTGGGTCAA3'
Nef,RP,(454-END)		5'GTCGACGCGCAGTCTTTGTAATACTCCGGA3'

The mutant Nef was constructed by using primer sequence as given in table (2). For making mutated Nef construct, the Nef gene was first amplified by primer FNef and RNef (mut) which amplified N-terminal and another amplification of Nef by using primer FNef (mut) and R Nef which amplified C- terminal of Nef. Now these two amplified product were used as template and further amplified by using forward FNef and reverse primer RNef. This amplified product was cloned in pEYFP-N1. Mutation at the desired site was confirmed by DNA sequencing.

### Mammalian Two Hybrid Assay

HEK-293 cell were seeded in 24 well plates (1×10^5^/well). After 24 hrs cells were co-transfected with ASK1 and Nef constructs along with pG5LUC vector. 0.75µg of each vector was transfected using transfection reagent ExGen 500. Cells were harvested after 48 hrs of transfection and lysed in 1x passive lysis buffer (Promega). Dual-Luciferase Reporter Assay System (DLR) (Promega E1910) was used in combination with a luminometer (Barthold), to assay both firefly luciferase and Renilla luciferase enzyme activities in transient transfected cell lysate sample.

### Annexin–V and PI staining

Apoptosis /necrosis index was evaluated using BD Annexin–V/PI apoptosis detecting kit following manufacturer’s protocol. Briefly cells were harvested after 48 hrs of transfection followed by washing twice with cold PBS and re-suspending in binding buffer at a concentration of 1x10^5^ cells/ml. Thereafter, 5 µl of FITC conjugated Annexin-V and 5 µl PI were added to the 100 µl aliquot of the cell suspension. The sample was gently vortexed followed by incubation for 15 min at RT (25°C) in the dark. After adding 400 µl of 1X binding buffer samples were subjected to flow cytometry in BD FACS Calibure system (USA). Cells scoring positive for Annexin-V but negative for PI were considered apoptotic and the resultant data was analyzed using CellQuesecPro software.

### Western Blot Analysis

Control, ASK1 and Nef transfected HEK-293 cell and Jurkat T cell were lysed in radio-immunoprecipitation assay (RIPA) buffer containing protease and phosphatase inhibitors (1mM phenylmethylsulfonyl fluoride, 10mg/ml aprotinin, and 10mg/ml leupeptin, 10µM sodium orthovanadate). Thereafter lysates were centrifuged at 13,000 × *g* at 4°C for 30 min. Protein content in the supernatants was determined using Folin Lowry’s method (1951). Cell lysate supernatants equivalent to 100 µg of protein were resolved through 12% SDS-PAGE and were transferred to PVDF (Millipore, USA) membranes. After blocking with 5% BSA in PBS containing 0.2% Tween-20, membranes were incubated at 4°C overnight with the respective antibodies. Blots were then incubated for 1 h at room temperature with horseradish peroxidase-conjugated secondary antibody (1:2000) and the peroxidase activity was analyzed with the ECL chemiluminescence substrate system (USA).

### Peptide synthesis

Peptide was synthesized manually via the solid phase method on Rink amide MBHA resin utilizing standard Fmoc chemistry. Cleavage of the peptide from the resin, and their precipitation was done by standard procedures. The peptide was purified by reverse phase HPLC on an analytical Waters Symmetry C18 column (300 Å, 5.0 µm, 4.6 mm × 250 mm) using a linear gradient of 20–80% acetonitrile for 40 min with a flow rate of 1.8 mL/min. Both Acetonitrile and water contained 0.1% trifluoroacetic acid. The purity of the peptides was further determined to be ≥95 by reverse phase analytical chromatography. Experimental molecular mass of the peptides was evaluated by MALDI-TOF analysis which was very close to that calculated mass.

### Peptide internalization study

Successful internalization of peptide was ascertained by treating HEK-293 cells with Alexa Fluor^555^ conjugated peptide. Briefly, 1x10^5^ cells were seeded on sterile, polylysine coated glass coverslips. After 24 hrs cells were treated with Alexa Fluor^555^ conjugated with Peptide-3. Cells were incubated for further 24 hrs followed by stain with DAPI and Alexa Fluor^488^ conjugated phallodion actin (F-Actin). Fluorescence microscopy was done using Zeiss LSM Image viewer software.

### In situ Proximity Ligation Assay:

For detecting interaction of Nef with wild type endogenously expressed ASK1 we employed the recently developed proximity ligation (PLA) assay for highly specific *in situ* detection coupled to a localized amplification reaction [38]. By allowing the detection of individual protein events such protein interactions, directly in individual cells and tissues, PLA techniques reveals dynamic interplay of molecules and their response to stimulus [39-41]. The proximity ligation assay depends on dual proximal binding by pairs of detection reagents to generate amplifiable DNA strands, which then serve as surrogate markers for the detected protein molecules. Soderberg et al., (2006) combined proximity ligation with rolling-circle amplification (RCA) for localized readout in fixed cells or tissues. Oligonucleotides attached on proximity probes i.e antibodies against two target proteins, when brought into close proximity by binding to adjacent proteins, serve as templates and guide the formation of circular DNA strands. Signal generated due to localized rolling-circle amplification (RCA), allows individual interacting pair of protein molecules to be visualized in cell lines and tissues. The assay was carried out using Duolink PLA^555^ kit in accordance with manufacturer’s (Olink biosciences, Uppsala, Sweden) protocol. Briefly, 2 ×10^5^ HEK-293 cells were seeded onto sterile, polylysine (3% of polylysine) coated glass cover slips. 48 hours post transfection (Nef plasmid), the endogenous ASK1 activity was stimulated using TNF-α. After 48 hrs cells were fixed in 3.7% buffered paraformaldehyde for 20 min in CO_2_ incubator. Thereafter cells were washed with DPBS at 4°C, permeablized with 0.5% triton×100 for 5 min. After washing with 1×PBS, blocking was carried out using 3% BSA/PBST. Incubation with anti-ASK1 mouse IgG and anti-Nef rabbit IgG was carried out overnight at 4°C at antibody dilution of 1:1000 and 1:50 respectively. Samples were then incubated with oligonucleotide labeled anti-mouse and anti-rabbit secondary antibodies (PLA probes) for one hour in preheated humidity chamber. After washing twice with wash buffer A (0.01M Tris, 0.15M NaCl and 0.05% Tween 20) 360 µl of ligation mixture (14 µl ligase, 112 µl ligase solution, 234 µl extra pure water) was added to each sample followed by incubation for 30 minutes in preheated humidity chamber. Thereafter ligation solution was tapped off and 360 µl amplification solutions (7µl polymerase, 112 µl 1× amplification stock and 241 µl extra pure water) was added. Samples were incubated in preheated humidity chamber for 100 min. Finally after washing twice with wash buffer A (0.2M Tris and 0.1M NaCl), cover slips were dried briefly and mounted in aqueous mounting media containing DAPI for visualization in Zeiss AxioImager.M2 fluorescent microscope (Carl Zeiss) with 63X, 1.4 oil emersion objectives.

### Statistical Analysis

Numerical data was tested for statistical significance using paired student’s t test (two-tailed). Differences were considered significant when p< .05(*), and very significant when p ≤ 0.01(**), and extremely significant when p ≤ 0,001(***). All data was analyzed by graphpad-prism 5.

## Conclusion

HIV-1Nef interacts with ASK1 through its C terminal (152 DEVGEANN159) domain and ASK1 interact with Nef through its N-terminal (1-318 aa) and C-terminal (905-1051 aa) domains.

We have shown HIV-1Nef potentiated Ser^967^ phosphorylation and rendered ASK1 functionally inactive and peptide designed by us successfully competed with HIV-1Nef for interaction with ASK1.

## Supporting Information

Figure S1 ASK1 (1-1051) fragment induce apoptosis through p38 phosphorylation. ASK1 (1-1051) transfected with and without Nef and cell were harwested after 48 hrs of transfection for analysis of p^38^ phosphorylation by western bloting. Densitometry analysis shows that ASK1 (1-1051) transfected cell showing more phosphorylation of p^38^ than ASK1 (1-1051) Nef cotransfected cell.
(DOC)

Figure S2 Deletion of N-terminal (1-345) and C-terminal (904-1051) of ASK1 (1-1051) causes loss of antiapoptotic function of HIV-1 Nef. ASK1(1-1051), ASK1(319-1051), ASK1(1-904), ASK1(319-904) of ASK1 different fragment were transfected with/without Nef showing 44.45%, 18.79%, 42.53% ,33.30% 46.35%, 28.04%, 30.12%, 30.03% respectively.this result indicate that on deletion of N-terminal or C-terminal of ASK1(1-1051) reduce antiapoptotic function of Nef.
(DOC)
