# Supplementary material for: Dynamics of Physical Interaction between HIV-1 Nef and ASK1: Identifying the Interacting Motif(S)
Source: PLoS One. 2013 Jun 14;8(6):e67586. doi: 10.1371/journal.pone.0067586 (PMC3683068; doi:10.1371/journal.pone.0067586)
Supplement: Figure S1 — ASK1 (1-1051) transfected with and without Nef and cell were harwested after 48 hrs of transfection for analysis of p38 phosphorylation by western bloting. Densitometry analysis shows that ASK1 (1-1051) transfected cell showing more phosphorylation of p38 than ASK1 (1-1051) Nef cotransfected cell. (DOC) [file pone.0067586.s001.doc]

**S-1**

**
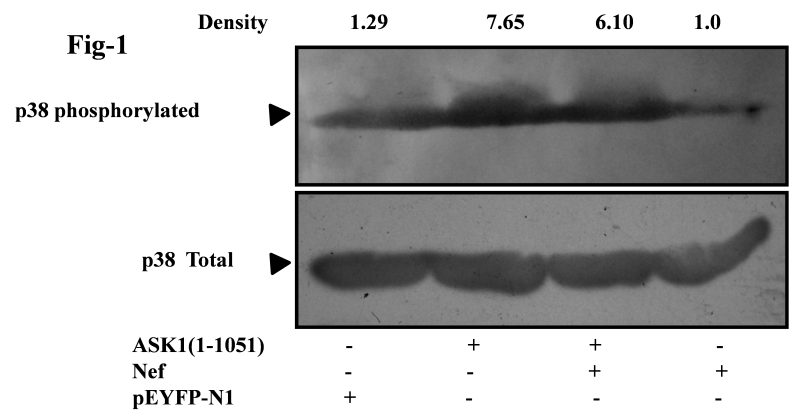
**

**ASK1 (1-1051) fragment induce apoptosis through p38 phosphorylation:** ASK1 (1-1051) transfected with and without Nef and cell were harwested after 48 hrs of transfection for analysis of p^38^ phosphorylation by western bloting. Densitometry analysis shows that ASK1 (1-1051) transfected cell showing more phosphorylation of p^38^ than ASK1 (1-1051) Nef cotransfected cell.
