# Supplementary material for: Dynamics of Physical Interaction between HIV-1 Nef and ASK1: Identifying the Interacting Motif(S)
Source: PLoS One. 2013 Jun 14;8(6):e67586. doi: 10.1371/journal.pone.0067586 (PMC3683068; doi:10.1371/journal.pone.0067586)
Supplement: Figure S2 — ASK1(1-1051), ASK1(319-1051), ASK1(1-904), ASK1(319-904) of ASK1 different fragment were transfected with/without Nef showing 44.45%, 18.79%, 42.53% ,33.30% 46.35%, 28.04%, 30.12%, 30.03% respectively.this result indicate that on deletion of N-terminal or C-terminal of ASK1(1-1051) reduce antiapoptotic function of Nef. (DOC) [file pone.0067586.s002.doc]

**S-2**

**Deletion of N-terminal (1-345) and C-terminal (904-1051) of ASK1 (1-1051) causes loss of antiapoptotic function of HIV-1 Nef:** ASK1(1-1051), ASK1(319-1051) ,ASK1(1-904), ASK1(319-904) of ASK1 different fragment were transfected with/without Nef showing 44.45%, 18.79%, 42.53% ,33.30% 46.35%, 28.04%, 30.12% , 30.03% respectively.this result indicate that on deletion of N-terminal or C-terminal of ASK1(1-1051) reduce antiapoptotic function of Nef.
